# QT Measurement and Heart Rate Correction during Hypoglycemia: Is There a Bias?

**DOI:** 10.4061/2010/961290

**Published:** 2010-12-22

**Authors:** Toke Folke Christensen, Jette Randløv, Leif Engmann Kristensen, Ebbe Eldrup, Ole Kristian Hejlesen, Johannes Jan Struijk

**Affiliations:** ^1^Device R&D, Novo Nordisk, Brennum Park 20A1.105, 3400 Hillerød, Denmark; ^2^Department of Medical Informatics, Aalborg University, Frederik Bajers Vej 7D1, 9220 Aalborg, Denmark; ^3^Education Centre, Steno Diabetes Center, Niels Steensens Vej 2, 2820 Gentofte, Denmark

## Abstract

*Introduction*. Several studies show that hypoglycemia causes QT interval prolongation. The aim of this study was to investigate the effect of QT measurement methodology, heart rate correction, and insulin types during hypoglycemia. *Methods*. Ten adult subjects with type 1 diabetes had hypoglycemia induced by intravenous injection of two insulin types in a cross-over design. QT measurements were done using the slope-intersect (SI) and manual annotation (MA) methods. Heart rate correction was done using Bazett's (QTcB) and Fridericia's (QTcF) formulas. *Results*. The SI method showed significant prolongation at hypoglycemia for QTcB (42(6) ms; *P* < .001) and QTcF (35(6) ms; *P* < .001). The MA method showed prolongation at hypoglycemia for QTcB (7(2) ms, *P* < .05) but not QTcF. No difference in ECG variables between the types of insulin was observed. *Discussion*. The method for measuring the QT interval has a significant impact on the prolongation of QT during hypoglycemia. Heart rate correction may also influence the QT during hypoglycemia while the type of insulin is insignificant. Prolongation of QTc in this study did not reach pathologic values suggesting that QTc prolongation cannot fully explain the dead-in-bed syndrome.

## 1. Introduction

The introduction of human insulin in the 1990s led to an increase in the number of sudden nocturnal deaths of young people with type I diabetes. This specific type of death in diabetes was termed the “dead in bed” syndrome and it was hypothesized that the deaths were caused by hypoglycemia [1]. The pathophysiological mechanisms behind the deaths are still not understood although circumstantial evidence suggests that they are cases of fatal cardiac arrhythmia. The proposed proarrhythmic effect of hypoglycemia is thought to be mediated by sympathoadrenal activation and hypokalaemia [2]. 

It has been reported that insulin-induced hypoglycemia affects repolarization of the cardiac cells in both healthy subjects [3, 4] and people with diabetes [5–7]. The altered repolarization is notable on the electrocardiogram (ECG) as a flattened T wave [4, 6] and a prolonged heart rate-corrected QT interval (QTc) [3, 5]. A prolonged QTc is associated with an increased risk of sudden cardiac death [8, 9], and QTc has thus been the primary variable investigated in studies of the proarrhythmic effect of hypoglycemia. The degree of QTc prolongation during clamped hypoglycemia ranges from 5 ms [6] to 60 ms [3] with one study reporting a prolongation of 156 ms [5]. Thus, in some studies the prolongation of QTc is seen to be insignificant while other studies show a significant and potentially dangerous prolongation. We hypothesize that differences in methodology when measuring the QT interval and heart rate correcting the QT interval may partly explain the discrepancies between the reported prolongations of QTc. It is known that Bazett's formula for heart rate correction is associated with both over- and undercorrection of QTc at heart rates outside a narrow range [10]. Nevertheless, Bazett's formula is still the most often used heart rate correction, when investigating QTc during hypoglycemia. The methodology when measuring the T wave offset also has an impact on the QT interval as for example the often used tangent method is more sensitive to the flattening of the T wave seen during hypoglycemia [11]. In addition, modern insulin analogues may have a different effect on the QTc than human insulin, although this has previously been shown not to be the case [12]. 

The “dead in bed” syndrome and the potential prolongation of QTc during hypoglycemia are still of concern to many patients and physicians. If these tragic deaths are to be avoided, an increased understanding of the phenomenon is necessary. It has been suggested that if patients with an increased risk of prolonged QT during hypoglycemia could be identified, a selective beta blocker might have a therapeutic role [2]. However, before considering preventive treatment, it is important to clarify if hypoglycemia indeed causes significant prolongation of QTc and identify the factors in study methodology that may cause the discrepancies among the reported results in the literature. Thus, in the present study, we investigate the potential bias associated with measurement technique, heart rate correction, and type of insulin when measuring the QTc during hypoglycemia.

## 2. Methods

### 2.1. Subjects

The study population consisted of 10 subjects (6 men, 4 women; age: 32 ± 9 years) with type 1 diabetes (C-peptide negative). Subjects had HbA1c <10%, duration of diabetes of 15 ± 10 years, and none had signs of neuropathy. Each subject was studied on two weekends separated by at least 1 month. At each weekend hypoglycemia was induced two times: saturday at 2 AM and 10 PM. Subjects were randomized to use either insulin aspart (Iasp) (NovoRapid, Novo Nordisk A/S, Denmark) or human insulin (HI) (Actrapid, Novo Nordisk A/S, Denmark) the first weekend in a cross-over design so the other type of insulin was used at the next weekend. Written informed consent was obtained from all subjects, and the study protocol was approved by the Regional Ethics Committee.

### 2.2. Procedures

A catheter (Venflon, Viggo AB, Sweden) was inserted into an antecubital arm vein for administration of insulin and glucose. Hypoglycemia was induced by a single bolus of insulin (0.1 U/kg bodyweight) injected directly into the blood. Blood glucose was measured by a HemoCue Analyzer (HemoCue AB, Angelholm, Sweden). Measurements of blood glucose were taken 30 minutes prior to insulin injection and at least every 5 minutes following the injection. When blood glucose <2.5 mmol/L was reached, intravenous glucose (10%) was administered to restore the blood glucose.

### 2.3. ECG Measurements

ECG was recorded from lead II by disposable Ag/AgCl electrodes (Blue Sensor L, Ambu A/S, Denmark). ECG was sampled by a data acquisition system (Portilab 16 + 2, Twente Medical Systems International, Holland) at 400 Hz with 12-bit resolution. 

**Figure 1:**
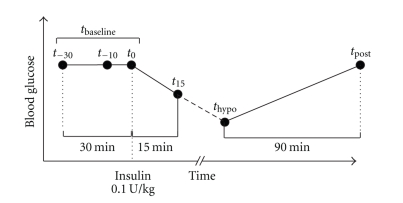
Schematic design of the study. Measurements of ECG (•) are taken three times in a baseline interval prior to insulin injection (*t*_−30_, *t*_−10_, and *t*_0_), 15 minutes after insulin injection (*t*_15_), at blood glucose nadir (*t*_hypo_), and 90 minutes after blood glucose nadir (*t*_post_).

Epochs of 60 seconds of ECG were analyzed 30 minutes prior to insulin injection (*t*_−30_), 10 minutes prior to insulin injection (*t*_−10_), at insulin injection (*t*_0_), 15 minutes after insulin injection (*t*_15_), at blood glucose nadir (*t*_hypo_), and 90 minutes after blood glucose nadir (*t*_post_) (Figure 1). QT intervals were measured from each epoch using both a manual annotation (MA) and a semi-automatic “slope intersect” (SI) method. 

MA measurements were carried out by independent QT experts (Spacelabs Healthcare, Washington, USA) blinded to the study design and all other information except the ECG. Each epoch was manually reviewed for artifacts and a representative beat was obtained. Distortion of the representative beat was minimized by selecting areas of the epoch with minimal artifacts to include in representative beat generation. From the representative beat QT intervals were measured from the first high-frequency deflection of the QRS complex to the offset of the T wave. Average RR interval of the QRS complexes selected for the representative beat was used to provide heart rate correction. In addition, the R peak and T peak amplitudes relative to the isoelectric line were measured. All measurements were done manually using an electronic caliper and reviewed by a cardiologist.

SI measurements were carried out using custom analysis software developed in MatLab (Version 7.8.0.347, The Mathworks, Inc., Natick, MA, USA). Templates representing the average PQRST complex in epochs were generated using manually selected areas with minimal artifacts. The end of the T wave in each template was determined automatically using the “slope intersect" method [13]. In addition, the R peak and T peak amplitudes relative to the isoelectric line were also measured. All templates and associated fiducial points were manually reviewed on-screen in random order by an observer blinded to the corresponding blood glucose and the subjects' clinical data. Templates were rejected if artifacts precluded reliable measurements but no adjustments to the QT interval were made to reduce the subjectivity of the measurements. The median RR interval in each segment was used to heart rate correcting the QT interval. In both MA and SI methods QT intervals were corrected by Bazett's formula (QTcB) [14] and Fridericia's formula (QTcF) [15]. As a measure of the T wave flatness the T peak to R peak amplitudes ratio (T/R Ratio) was calculated.

### 2.4. Data Analysis

Measurements at *t*_−30_, *t*_−10_ and *t*_0_ were averaged and collectively called *t*_baseline_ to reduce the amount of statistical analyses. A linear mixed effects model was used to analyze the changes in ECG variables. Time (levels: *t*_baseline_, *t*_15_, *t*_hypo_, and *t*_post_) and treatment (levels: Iasp, HI) were included as fixed effects in the model together with a time-treatment interaction term. Subjects were included in the model as a random effect on the intercept. Weekend was included as a random effect within subjects and episodes as a random effect within weekends. Separate models for each ECG variable were fitted using restricted maximum likelihood. An analysis of variance of the fitted model was used to test for significant changes in variables. With variables showing statistical significance, Dunnett's posthoc tests were used to test for significant differences between different factor levels. *P* values <.05 were considered significant. Results from the statistical model are reported as mean (SE), all other results are reported as mean ± SD. Statistical analyses were performed in R version 2.9.1.

## 3. Results

Seven (17.5%) of the recorded episodes of hypoglycemia were excluded because the subject had a blood glucose ≤3.5 mmol/L at *t*_−30_ (*n* = 6) or because of instrumentation issues (*n* = 1). Thus a total of 33 episodes were used in the data analysis with a minimum of two episodes available from each subject. Hypoglycemia was reached 45 ± 32 minutes after insulin was administered with blood glucose at nadir of 2.4 ± 0.3 mmol/L. The measured variables summarized for each time point are shown in Table 1.

Using the MA method significant prolongation of QTcB from *t*_baseline_ to *t*_hypo_ was observed (ΔQTcB: 7(2); *P* < .05) but no prolongation of QTcF was seen (ΔQTcF: 1(2); *P* > .05). With the SI method both QTcB and QTcF prolonged significantly (ΔQTcB: 42(6); *P* < .001, ΔQTcF: 35(6); *P* < .001) (Table 2). Heart rate and T/R ratio decreased significantly with both the MA and SI methods (*P* < .001). The changes in variables from *t*_baseline_ to *t*_15_ were similar to the change from *t*_baseline_ to *t*_hypo_ (Table 2). At *t*_post_, QTcB and QTcF had returned to *t*_baseline_ levels while RR interval and T/R ratio remained decreased for both MA and SI methods (Table 2). 

There was a significant difference between ΔQTcF measured by the SI and MA methods (34 ms; *P* < .001) (Table 3, Figure 3). The difference between ΔQTcB and ΔQTcF was also significant (8 ms; *P* < .001) (Table 3, Figure 4). No significant difference between HI and Iasp was found on any of the measured ECG variables.

## 4. Discussion

The MA method showed modest increase in QTcB and no increase in QTcF during hypoglycemia, while the SI method showed considerable prolongation of both QTcB and QTcF. Comparing the two methods directly showed that SI underestimated the QT interval at baseline and overestimated it at hypoglycemia compared to MA.

**Figure 2:**
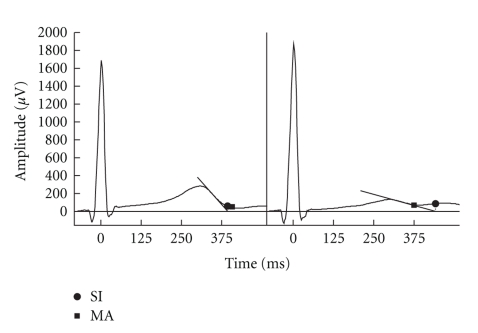
Measurements of the T wave offset from ECG lead II using the semiautomatic slope-intersect method (SI) and manual annotation (MA) method. The two ECGs are from *t*_0_ (left side) and at *t*_hypo_ (right side) at the same episode. At *t*_0_ the SI method underestimates the end of the T wave compared with the MA method. With the flattening of the T wave at *t*_hypo_ the SI method overestimates the end of the T wave compared with the MA method.

**Figure 3:**
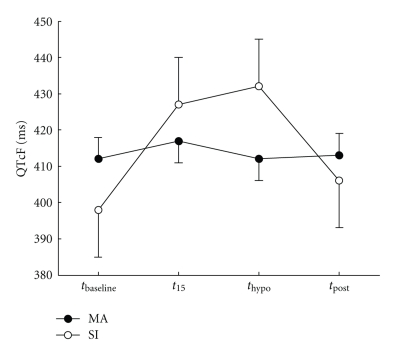
The difference over the course of a hypoglycemia episode in QTc corrected by Fridericia's formula (QTcF) for the manual annotation (MA) and slope-intersect (SI) methods. Data is mean ± SE estimated from the statistical model.

**Figure 4:**
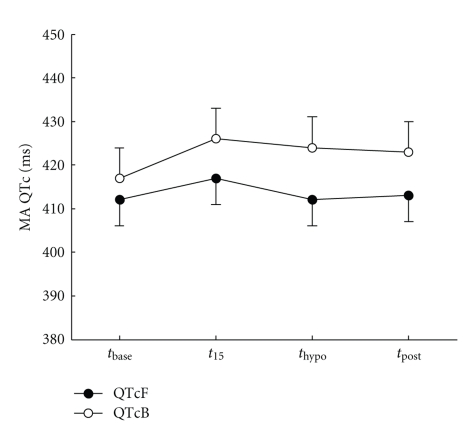
Difference between QTc corrected by Bazett's (QTcB) and Fridericia's (QTcF) formulas. The QT interval is measured using the manual annotation (MA) method. Data is mean ± SE estimated from the statistical model.

The use of the SI method for measuring the end of the T wave is known to be sensitive to changes in T wave amplitude, although the method was originally meant for cases with partial T-U fusion [11, 16]. In particular a flattened T wave will cause an overestimation of the QT interval with the SI method when compared to the MA method (Figure 2). Since there exists no gold standard of measuring the QT interval, neither of the measurement techniques can from this study be judged more correctly than the other. However, the discrepancy between the two methods illustrates that comparing studies of hypoglycemia using different QT measurement methods may be problematic. Also, it is apparent from the results that the SI method produces significantly longer QT intervals than the MA method which could indicate a higher probability of false positives with the SI method. An approach which could eliminate the bias associated with measuring the QT interval is to use alternative T wave morphology variables. Xue and Reddy [17] used principal component analysis of the T wave and showed that this approach had superior reproducibility than several QT measurement methods. Alternative T wave morphology parameters might therefore be better at characterizing changes in repolarization during hypoglycemia.

A pathologically prolonged QTc interval is usually defined as >450 ms for men and >470 ms for women [18]. In the present study, mean QTc did not exceed these thresholds with any of the methods. This could indicate that prolongation of the QTc cannot in itself explain the mechanism implicated in the dead-in-bed syndrome. 

The differences between QTcB and QTcF in this study were larger at hypoglycemia than at baseline. It is known that Bazett's formula tends to overcorrect the QTc at higher heart rates [10]. In this study we observed a significant increase in heart rate during hypoglycemia which may have contributed to an overcorrection by Bazett's formula compared to Fridericia's formula. Similar findings of differing results using the two correction formulas have been reported [6] although they in other cases produce similar results [19]. 

One of the main limitations of the study is the absence of a control group. Without a control group it is less clear if the observed QT prolongation is caused by hypoglycemia per se. Indeed, insulin could act as a confounding variable as it has been shown to cause moderate QT interval prolongation [20]. To account for the effect of insulin, we measured the ECG variables of interest 15 minutes after insulin injection where the subjects were still normoglycemic. We anticipated that this measurement would quantify the effect of hyperinsulinemia alone. The results show that the change in ECG variables 15 minutes after insulin injection is comparable to the change at hypoglycemia. This could indicate that the observed changes during hypoglycemia may not be caused by hypoglycemia per se but rather by hyperinsulinaemia. We acknowledge that the small number of subjects in the study limits its generalisability to the general population, although measurements on each subject were repeated to reduce intrasubject variation. Additionally, the use of only one ECG lead for QT measurement may have introduced some variation in the measurements, which could have been mitigated by the use of several leads. 

The results of this study are in agreement with previous studies of experimentally induced hypoglycemia using the MA method [4, 6] although some studies also report a QTc prolongation [5, 11]. Studies using the SI method consistently find significantly prolonged QTc during hypoglycemia [2, 3, 12, 19, 21]. We found no difference in the observed variables between HI and Iasp which is in line with previous findings [12]. Ireland and colleagues [11] compared the SI and MA methods and concluded that the SI method was preferred over the MA method because of a lower inter observer difference despite an overestimation of the QT interval at hypoglycemia. We cannot infer on inter-observer differences from our study but our results confirm that the SI method overestimates the QT interval during hypoglycemia.

## 5. Conclusion

Conclusively, our results suggest that the methodology used for measuring and heart rate correcting the QT interval during hypoglycemia may have a significant impact on the measured prolongation of QTc. The SI method overestimates the QT interval compared to the MA method at hypoglycemia while Bazett's formula overcorrects the QTc compared to Fridericia's formula. The type of insulin for inducing hypoglycemia does not influence QT prolongation. Prolongation of QTc in this study did not reach pathologic values which suggest that additional factors play a role in the pathogenesis of the dead-in-bed syndrome.

## Figures and Tables

**Table 1:** Blood glucose and ECG variables at baseline (*t*_base_), at blood glucose nadir (*t*_hypo_) and 90 minutes after blood glucose nadir (*t*_post_). Results are mean ± SD [range].

Method	Variable	Time		
		*t*_base_	*t*_hypo_	*t*_post_
	Blood Glucose	10.4 ± 3.8 [5.0–19.5]	2.4 ± 0.3 [1.7–2.9]	10.1 ± 3.3 [5.7–18.7]
MA	RR (ms)	933 ± 157 [665–1278]	845 ± 128 [640–1125]	871 ± 159 [665–1245]
	QTcF (ms)	412 ± 19 [379–463]	412 ± 21 [374–460]	413 ± 22 [378–453]
	QTcB (ms)	418 ± 24 [384–472]	425 ± 23 [391–475]	424 ± 26 [375–473]
	T/R Ratio (−)	0.3 ± 0.14 [0.1–0.62]	0.2 ± 0.1 [0.03–0.44]	0.23 ± 0.11 [0.07–0.51]

SI	RR (ms)	935 ± 160 [658–1272]	836 ± 139 [625–1132]	869 ± 161 [637–1249]
	QTcF (ms)	399 ± 27 [363–452]	433 ± 61 [368–501]	408 ± 35 [359–496]
	QTcB (ms)	405 ± 32 [364–467]	447 ± 66 [379–517]	419 ± 40 [364–506]
	T/R Ratio (−)	0.31 ± 0.13 [0.12–0.66]	0.2 ± 0.1 [0.07–0.47]	0.24 ± 0.1 [0.12–0.47]

**Table 2:** Estimated changes in ECG variables over time with *t*_base_ as reference point. Estimates are based on a statistical model fit to each variable.

		Time		
	
*t*_15_	*t*_hypo_	*t*_post_
Method	Variable	*β* (SE) 95% CI	*β* (SE) 95% CI	*β* (SE) 95% CI
MA	RR (ms)	−55 (16)^† ^[−92;-18]	−87(14)^‡^ [−121;−53]	−64(15)^‡ ^[−99;−29]
	QTcF (ms)	5 (2) [−1;10]	1(2) [−4;5]	1(2) [−4;6]
	QTcB (ms)	QTcB (ms)	9 (3)^† ^[3;15]	7(2)^†^ [2;13]	6(2)^† ^[1;12]
	T/R Ratio (−)	−0.07 (0.01)^‡ ^[−0.1;−0.05]	−0.1(0.01)^‡^ [−0.12;−0.08]	−0.06 (0.01)^‡^ [−0.08;−0.04]

SI	RR (ms)	−60 (16)^‡ ^[−97; −23]	−98(14)^‡^ [−132;−64]	−69(15)^‡ ^[−105; −34]
	QTcF (ms)	30 (6)^‡ ^[15;44]	35(6)^‡^ [22;48]	8(6) [−5;22]
	QTcB (ms)	34 (7)^‡ ^[19;50]	42(6)^‡^ [28;56]	13(6) [−1;28]
	T/R Ratio (**−**)	−0.08 (0.01)^‡ ^[−0.1; −0.06]	−0.1(0.01)^‡ ^[−0.12; −0.08]	−0.06 (0.01)^‡ ^[−0.08; −0.04]

Significances (compared with *t*_baseline_): ^†^ *P* < .05, ^‡^ *P* < .001.

**Table 3:** Differences at *t*_base_ and *t*_hypo_ between the two methods for QT measurement (semi-automatic slope-intersect (SI) and manual annotation (MA)) and heart rate correction (Bazett's (QTcB) and Fridericia's (QTcF)).

		*t*_base_	*t*_hypo_	*t*-test (*t*_hypo_−*t*_base_)	
Difference	Method	Mean ± SD	Mean ± SD	Mean 95% CI	*P*-value
QTcB-QTcF	MA	6 ± 11	13 ± 10	7 [5;8]	<.001
	SI	6 ± 11	13 ± 11	8 [5;10]	<.001

SI – MA	QTcB	−13 ± 12	22 ± 51	35 [20;51]	<.001
	QTcF	−13 ± 12	21 ± 50	34 [19;49]	<.001
